# The Impact of Diabetes Mellitus on Sensorineural Hearing Loss: A Cross-Sectional Study in Eastern India

**DOI:** 10.7759/cureus.52431

**Published:** 2024-01-17

**Authors:** Utkal P Mishra, Ganakalyan Behera, Anjan K Sahoo, Satyajit Mishra, Radhakant Patnaik

**Affiliations:** 1 Department of Otolaryngology - Head and Neck Surgery, All India Institute of Medical Sciences, Bhopal, Bhopal, IND; 2 Department of Otolaryngology - Head and Neck Surgery, Bhima Bhoi Medical College and Hospital, Balangir, Balangir, IND; 3 Department of Otolaryngology - Head and Neck Surgery, Veer Surendra Sai Institute of Medical Sciences and Research, Burla, IND

**Keywords:** hba1c levels, rural india, pure tone audiometry, audiometry, hearing loss, eastern india, sensorinerual hearing loss, type-2 diabetes mellitus

## Abstract

Objective

Hearing loss as a comorbidity of type 2 diabetes mellitus (type 2 DM) is frequently overlooked by patients and healthcare professionals because of a lack of awareness. This cross-sectional study aims to investigate the impact of DM on sensorineural hearing loss (SNHL) in the population of Eastern India. The primary objectives are to assess the prevalence and severity of SNHL among individuals with DM, explore demographic and clinical factors associated with hearing impairment, and contribute valuable insights to the understanding of this relationship in a specific regional context.

Methods

An institutional-based cross-sectional study was conducted on 198 patients with type 2 DM. Of these, 46 patients were excluded based on exclusion criteria. All patients underwent detailed demographic and clinical assessments, including glycemic control, DM duration, and associated complications. Pure tone audiometry was used to evaluate hearing thresholds. Otoacoustic emission testing was performed to assess cochlear dysfunction.

Results

A high prevalence of SNHL (70.4%) was observed among the 152 participants meeting the inclusion criteria. Females exhibited a higher prevalence than males, and most participants experienced mild SNHL. Rural residence, lower socioeconomic status, and poor glycemic control were associated with increased SNHL. Significant associations were found between hearing loss severity and DM duration, glycosylated hemoglobin (HbA1c) levels, and complications. Among complications, a strong association was noted with diabetic neuropathy. No significant association was observed with the presence or absence of otoacoustic-emission.

Conclusion

This study reveals a substantial impact of DM on SNHL in Eastern India, emphasizing the importance of routine hearing assessments in diabetic populations. The findings contribute to regional understanding and have implications for targeted healthcare interventions and preventive strategies.

## Introduction

Type 2 diabetes mellitus (type 2 DM) is a global health concern with a rising prevalence, impacting millions worldwide. India is often referred to as the "diabetes capital of the world" because of its staggering diabetes burden [1]. In India, both urban and rural populations are affected, with a substantial increase in the prevalence of type 2 DM. The International Diabetes Federation has estimated that over 101 million adults in India live with diabetes, a number expected to rise significantly in the coming years [2]. The Eastern Indian states, a relatively underdeveloped corridor of the country, face unique socioeconomic and cultural aspects that influence diabetes risk given the rapid urbanization, shifts in dietary patterns, genetic predispositions, and low socioeconomic status.

While the well-established complications of DM include cardiovascular disease, kidney dysfunction, retinopathy, and neuropathy, emerging evidence suggests a potential link between DM and sensorineural hearing loss (SNHL). According to the World Health Organization, approximately 466 million people worldwide suffer from disabling hearing loss, while over one billion young adults are at risk of developing hearing impairment, marking it as a significant global health concern [3]. The complex interplay between diabetes and auditory function has become a subject of interest among researchers. However, the results of studies on diabetes and hearing impairment are not consistent. Although many acknowledge mild and subclinical SNHL in DM, some studies do not find any significant relationship between diabetes and increased risk of hearing loss. A major health survey in the United States revealed that all frequencies are affected by hearing impairment in diabetic patients, with high-frequency impairment being more prevalent than low/mid-frequency ranges [4]. Conversely, other studies suggest a primary impact on low frequencies [5]. Despite numerous studies demonstrating the connection between type 2 DM and hearing loss, there is limited awareness of hearing impairment as a potential comorbid condition among diabetic patients and healthcare professionals.

The purpose of this cross-sectional study was to provide insight into the prevalence of hearing loss in the diabetic population of Eastern India and to identify the potential risk factors associated with diabetes-induced auditory dysfunction.

## Materials and methods

This institutional-based cross-sectional study was conducted in the Department of Otorhinolaryngology - Head and Neck Surgery of a tertiary care hospital in Eastern India. It was reviewed and approved by the VIMSAR Institutional Human Ethics Committee, Burla, India, with LOP no. 011/043 before the initiation of the study. Written and informed consent were obtained from all study participants, and stringent measures were taken for the anonymization of collected data. Patients under 60 years of age diagnosed with type 2 DM were recruited through a nonrandomized convenience sampling method from the outpatient department. Patients with a history of loud noise exposure, ototoxic drug intake, history of ear discharge, smoking, head injury, and pregnancy were excluded from the study. The comprehensive inclusion and exclusion criteria are detailed in Table 1, providing a clear overview of the eligibility parameters for participants in the study. All patients were interviewed with a self-structured, self-administered questionnaire. Demographic details, duration and control of DM, family history, treatment history, history of complications related to diabetes, and any complaints regarding hearing are recorded. All patients then proceed for evaluation in the diabetes clinic, followed by an audiometric evaluation.

**Table 1:** Inclusion and exclusion criteria

	Criteria
Inclusion criteria	Patients under 60 years of age diagnosed with type 2 diabetes mellitus (type 2 DM).
Exclusion criteria	Patients with a history of loud noise exposure, ototoxic drug intake, history of ear discharge, smoking, head injury, and pregnancy. Patients with abnormal tympanometry findings suggestive of middle ear pathology.

Evaluation in a diabetes clinic

Routine blood investigations were conducted for glycosylated hemoglobin levels (HbA1c), fasting and postprandial blood glucose levels, blood urea, and serum creatinine levels. Initial diagnosis of type 2 DM was made according to the diagnostic criteria led by the American Diabetes Association (ADA) as fasting plasma glucose (FPG) level of 126 mg/dL (7.0 mmol/L) or higher, or two-hour plasma glucose level of 200 mg/dL (11.1 mmol/L) or higher [6]. Patients underwent fundoscopic evaluation by a trained ophthalmologist for evidence of diabetic retinopathy like macular edema, microaneurysms, retinal hemorrhage, cotton-wool spots, neovascularization, or retinal detachment. Diabetic nephropathy was diagnosed by persistent albuminuria (>300 mg/24 h) on two or more occasions or an albumin-to-creatinine ratio (ACR) of >300 mg/g [6]. Patients also underwent neurological evaluation for evidence of diabetic neuropathy.

Audiometric evaluation

An otoscopic examination was done, and any wax or debris in the ear canal was cleared. Tympanometry was performed before audiometric evaluation to exclude any preexisting middle ear pathology. Only patients with “type-A tympanogram,” suggesting a normal middle ear function, were included in the study. Pure tone audiometry was performed for air and bone conduction thresholds with Interacoustics^TM^ AD226 audiometer (Interacoustics A/S, Middelfart, Denmark), including 250, 500, 1000, 2000, 4000 and 8000 Hz frequencies. The audiometric average was obtained from 500, 1,000, and 2,000 Hz frequencies. An average audiometric threshold of less than 25dB in the worst ear is considered to be “no hearing impairment.” Hearing loss was classified into mild (26-40 dB), moderate (41-60dB), severe (61-80dB), and profound (≥81dB) hearing loss as per the World Health Organization grading of hearing impairment [7]. Otoacoustic emission was performed as transient evoked (TEOAE) and distortion product (DPOAE), and results were recorded as “PASS” or “REFER” for presence or absence respectively.

Statistical analysis

Statistical analyses were performed using Statistical Product and Service Solutions (SPSS) (version 29.0; IBM SPSS Statistics for Windows, Armonk, NY). All categorical variables were presented as numbers and percentages, while continuous variables were expressed as mean ± standard deviation (SD). Differences were analyzed by t-test for quantitative variables and chi-square test for categorical variables. A P value of <0.05 was considered statistically significant.

## Results

Out of the initial pool of 198 patients diagnosed with type 2 DM, 46 individuals were excluded from the study. This was based on predefined exclusion criteria: 12 patients with a history of ear discharge, 11 patients with a documented history of loud noise exposure, 10 patients with a history of ototoxic drug intake, 10 patients who reported smoking, and three patients with abnormal findings in tympanometry. These exclusions were crucial to ensure that the study focused on a cohort with a final sample size of 152 without any confounding factors that could impact the assessment of hearing loss associated with DM. Out of 152 patients, 85 (55.9%) were male, and 67 (44.1%) were female. The mean age of the study population was 49 years, ranging from 44 to 58 years. Most of the study participants were rural, and the majority belonged to a lower socioeconomic status (Table 2).

**Table 2:** Demographic profile of the study population

Characteristics	n = 152	%
Gender		
Male	85	55.9
Female	67	44.1
Age (in years)	49.0 (44.0-58.0)	
Residency		
Urban	28	18.4
Rural	124	81.6
Socioeconomic status		
Upper	12	7.9
Middle	54	35.5
Lower	86	56.6

Most of the patients have a positive family history of diabetes, a duration of more than ten years, and poor glycemic control. Almost 40 percent of the study population have no hearing complaints. Among the complications associated with DM, diabetic neuropathy was found to be the most prevalent, followed by diabetic nephropathy (Table 3). A positive history of hypertension was found in 95% of patients (n=144), but, on examination, blood pressure was found to be controlled with antihypertensive medications. None of the patients had a history of coronary artery disease.

**Table 3:** Clinical characteristics of the study population DM: Diabetes mellitus, HbA1c: glycosylated hemoglobin, (6-7% = good glycemic control, 7-8% = moderate glycemic control, ≥ 8% = poor glycemic control)

Characteristics	n = 152	%
Family history of DM		
Present	117	76.9
Absent	35	23.1
Duration of diabetes		
< 5 yrs	35	23.0
6-10 yrs	27	17.8
> 10 yrs	90	59.2
Self-perceived hearing loss		
Present	92	60.6
Absent	60	39.4
HbA1c		
6-7%	15	9.9
7-8%	58	38.2
≥ 8%	79	51.9
Complications of DM		
Retinopathy	26	17.1
Nephropathy	38	25.0
Neuropathy	57	37.5

Table 4 illustrates the association of clinical and demographical variables with the severity of hearing impairment. The prevalence of hearing loss in type 2 DM was found to be 70.4%. Of the 152 patients, SNHL was found to be more prevalent in females (74.7%) than males. Most of the patients had mild SNHL. Among diabetic patients without any hearing-related complaints, mild SNHL was found in 25% of the cases. In evaluating the hearing loss pattern among the diabetic population, a predominant high-frequency involvement was observed in 67% of the cases. The remaining patients exhibited involvement across all frequencies, as revealed by pure tone audiometry. The majority of the patients with hearing loss were rural and belonged to lower socioeconomic status. Hearing loss was more prevalent in patients with poor glycaemic control (HbA1c ≥ 8%) compared to those with good glycaemic control (Table 4). Moreover, profound hearing loss was seen only in patients with moderate and poor glycemic control. SNHL was more prevalent in patients with diabetic neuropathy. A statistically significant association was observed between the severity of hearing loss with the duration of DM, HbA1c levels, and complications of DM. On otoacoustic-emission testing, DPOAE was absent in 91% of diabetics with hearing loss. However, no significant association was observed between DPOAE in diabetics with hearing loss and those with normal hearing groups (Table 5).

**Table 4:** Association of demographic and clinical variables with audiometric findings of the study population The data are presented in the form of n (%). HbA1c: glycosylated hemoglobin

Variables	No hearing loss	Mild SNHL	Moderate SNHL	Severe to profound SNHL	P value
Gender					0.563
Male	28 (32.9)	34 (40.0)	20 (23.5)	3 (3.5)	
Female	17 (25.3)	33 (49.2)	16 (23.9)	1 (1.4)	
Geographic area					0.006
Urban	2 (7.1)	14 (50.0)	12 (42.8)	0 (0)	
Rural	43 (34.7)	53 (42.7)	24 (19.3)	4 (3.2)	
Socioeconomic status					0.587
Upper	3 (25.0)	6 (50.0)	2 (16.6)	1 (8.3)	
Middle	17 (31.5)	26 (48.1)	11 (20.4)	0 (0)	
Lower	25 (29.1)	35 (40.7)	23 (26.7)	3 (3.5)	
Family history of diabetes					0.010
Present	27 (23.1)	55 (47.0)	31 (26.5)	4 (3.4)	
Absent	18 (51.4)	12 (34.3)	5 (14.3)	0 (0)	
Duration of diabetes					0.0054
< 5 yrs	20 (57.1)	12 (34.3)	3 (8.6)	0 (0)	
6- 10 yrs	6 (22.2)	13 (48.1)	7 (25.9)	1 (3.7)	
> 10 yrs	19 (21.1)	42 (46.7)	26 (28.9)	3 (3.3)	
Self-perceived hearing loss					3.073
Present	0 (0)	52 (56.5)	36 (39.1)	4 (4.3)	
Absent	45 (75.0)	15 (25.0)	0 (0)	0 (0)	
HbA1c					0.0047
6-7%	9 (60.0)	5 (33.3)	1 (6.66)	0 (0)	
7-8%	22 (37.9)	26 (44.8)	8 (13.8)	2 (3.4)	
≥ 8%	14 (17.7)	36 (45.6)	27 (34.2)	2 (2.5)	
Complications of diabetes					0.0022
Retinopathy	5 (19.2)	15 (57.8)	5 (19.2)	1 (3.8)	
Nephropathy	7 (18.4)	23 (60.5)	5 (13.1)	3 (7.9)	
Neuropathy	14 (24.6)	18 (31.5)	25 (43.9)	0 (0)	

**Table 5:** Association of hearing loss in type 2 DM with DPOAE The data are presented in the form of n (%). DPOAE: distortion product otoacoustic emission. "PASS" signifies the presence of DPOAE, and "REFER" signifies the absence of DPOAE.

Characteristics	DPOAE PASS	DPOAE REFER	
Diabetes with hearing loss (n=107)	9 (8.4)	98 (91.6)	p > 4.383
Diabetes with normal hearing (n=45)	42 (93.3)	3 (6.6)	

## Discussion

The results of this study involving 152 patients with type 2 DM provide valuable insights into the prevalence and associations of hearing impairment in this population.

Age, sex, and onset of SNHL

The mean age of the study population is 49 years. It indicates that in Eastern India, type 2 DM is more prevalent in relatively younger populations compared to other parts of the world. In many studies on hearing loss in DM, the mean age of the study population ranged from 50 to 60 years [8,9]. The lower age range in our study helped eliminate presbycusis as a potential confounding factor. SNHL is found to be more prevalent in the female population, which corroborates numerous previous studies [10]. In this study, the SNHL in all participants is of gradual onset. We did not find any patients with sudden SNHL in this study. However, many authors have reported sudden SNHL in diabetic patients in their previous studies [10]. The absence of sudden SNHL in our study highlights potential variations in the presentation of hearing issues among diabetic populations and highlights the importance of comprehensive audiological examination and understanding of diverse patterns within this context.

Prevalence of SNHL in type 2 DM

In this study, the prevalence of SNHL in type 2 DM is found to be 70.4%. This high prevalence is attributed to the relatively severe and long-standing diabetes in the study population. It may also be because of the willingness to undergo audiological evaluation by a majority of those patients who are already having hearing problems. An earlier study on type II DM in coastal India reported an even more staggering prevalence of 97.6% among the study participants [11]. Furthermore, a longitudinal population-based study involving 3,571 participants investigating the correlation between non-insulin-dependent DM (NIDDM) and hearing loss reported a 67.5% prevalence of hearing loss [12]. This indicates that DM acts as a risk factor for SNHL. Our analysis of pure tone audiometry thresholds revealed that mild SNHL is the predominant type, both among diabetics with reported hearing complaints and those without any complaints. A cross-sectional study conducted in the Saudi Arabian population also found a high prevalence of mild SNHL in the diabetic population [13]. SNHL in diabetes is because of chronic hyperglycemia and resultant microvascular damage leading to cochlear microangiopathy. This is reflected in this study as the most prevalent pattern of hearing loss observed in the study population was high-frequency SNHL suggesting cochlear microangiopathy. Additionally, diabetes-related inflammation and oxidative stress may contribute to the damage of the auditory nerves and hair cells. The cumulative effect of these processes results in SNHL, often manifesting initially in high frequencies.

Association of the HbA1c level with SNHL

HbA1c is clinically accepted as a more accurate reflection of plasma glycemic status over the previous two to three months, serving as a reliable diagnostic biomarker for Type 2 DM. A study conducted by Al-Rubeaan et al. demonstrated that individuals with poor glycemic control (HbA1c ≥ 8%) exhibited a higher prevalence of hearing loss compared to those with good glycemic control (HbA1c < 8%) [13]. Similar findings are also noted in our study, with a statistically significant prevalence of SNHL in HbA1c ≥ 8%. It reflects that poor glycaemic control contributes to more pronounced cochlear hair cell damage, primarily attributed to increased oxidative stress and inflammation. The distortion product otoacoustic emission represents a function of the outer hair cell of the cochlea. The absence of DPOAE signifies cochlear hair cell damage in SNHL. Notably, in this study, 91% of patients with hearing loss exhibited absent distortion product otoacoustic emissions (DPOAE), indicating a potential link to hyperglycemia-induced cochlear microangiopathy.

Association of complications of type 2 DM with SNHL

In the exploration of risk factors for the development of hearing loss in DM, we observed a strong association of three microvascular complications (i.e., retinopathy, nephropathy, and neuropathy with SNHL). These findings are consistent with many studies, notably involving neuropathy and nephropathy. Ren et al., in a cohort of 160 diabetic patients, analyzed hearing loss in type 2 DM and found a strong association of high-frequency SNHL with diabetic neuropathy [8]. Similarly, several animal studies have also observed the loss of spiral ganglion cells in diabetic mice [14,15]. This indicates a similar pathophysiology involving both neuropathy and cochlear hair cell damage. Similar pathophysiological processes involving electrolyte and fluid transportation in the stria vascularis and glomerulus indicate potential cochlear damage with compromised kidney function.

How to prevent the progression of hearing loss in type 2 DM

Preventing the progression of hearing loss associated with type 2 DM, akin to many microvascular complications of diabetes, is a plausible goal. These complications share common factors linked to aging and compromised microcirculatory blood flow. Adopting fundamental lifestyle modifications, particularly through heightened physical activity, has been shown to enhance microcirculation [16]. While the aging process is inevitable, proactively managing one's health by adhering to a nutritious diet, regulating blood glucose and blood pressure, and embracing regular physical activity can potentially mitigate the onset and advancement of various diabetes-related complications, including hearing loss.

Strengths and limitations of the study

In this study, efforts were made to eliminate potential confounding factors such as smoking, noise-induced hearing loss, ototoxicity, and presbycusis, all of which are recognized as exacerbating factors for hearing loss. This study has certain limitations that need mentioning. The sample size is relatively small, and the study population is not homogenous with respect to the severity and duration of diabetes. Nevertheless, the study successfully identified a high prevalence of hearing loss within an underprivileged population in Eastern India. Chronic hypertension is implicated as an important etiology of SNHL in some studies. Although a history of hypertension was noted in 95% of the study population, all patients were normotensive during clinical examination, as they were under antihypertensive medications. As a result, hypertension can be safely excluded as a confounding factor in this study.

## Conclusions

Hearing loss in diabetic patients is a notable concern in Eastern India. This study revealed that many diabetic patients without hearing complaints exhibited mild SNHL. Conversely, patients with hearing complaints lacked awareness of hearing loss in DM. Eastern India is characterized by a mix of rural and urban populations that are relatively underserved. Limited awareness, late diagnosis, and potential genetic factors may further exacerbate the impact of diabetes-related hearing loss in this population. Studying the dynamics of this correlation in Eastern India is essential for understanding regional nuances, informing targeted healthcare interventions, and implementing preventive strategies to address the specific challenges posed by diabetes-related hearing impairment in this geographic context. Periodic screening of hearing should be considered in the follow-up evaluation of patients with type 2 DM.

## References

[1] Mithal A, Majhi D, Shunmugavelu M, Talwarkar PG, Vasnawala H, Raza AS (2014). Prevalence of dyslipidemia in adult Indian diabetic patients: a cross-sectional study (SOLID). Indian J Endocrinol Metab.

[2] Kaveeshwar SA, Cornwall J (2014). The current state of diabetes mellitus in India. Australas Med J.

[3] Olusanya BO, Davis AC, Hoffman HJ (2019). Hearing loss: rising prevalence and impact. Bull World Health Organ.

[4] Bainbridge KE, Hoffman HJ, Cowie CC (2011). Risk factors for hearing impairment among U.S. adults with diabetes: National Health and Nutrition Examination Survey 1999-2004. Diabetes Care.

[5] Chen MY, Du YL, Zhu QR, Xie WQ, Zhang BD, Xu LW (2017). [The relationship between type Ⅱ diabetes mellitus and hearing loss: a meta-analysis]. Journal of Clinical Otorhinolaryngology, Head, and Neck Surgery.

[6] American Diabetes Association (2010). Diagnosis and classification of diabetes mellitus. Diabetes Care.

[7] Olusanya BO, Davis AC, Hoffman HJ (2019). Hearing loss grades and the international classification of functioning, disability and health. Bull World Health Organ.

[8] Ren H, Wang Z, Mao Z, Zhang P, Wang C, Liu A, Yuan G (2017). Hearing loss in type 2 diabetes in association with diabetic neuropathy. Arch Med Res.

[9] Calvin D, Watley SR (2015). Diabetes and hearing loss among underserved populations. Nurs Clin North Am.

[10] Kim MB (2017). Diabetes mellitus and the incidence of hearing loss: a cohort study. Int J Epidemiol.

[11] Dosemane D, Bahniwal RK, Manisha N, Khadilkar MN (2019). Association between type 2 diabetes mellitus and hearing loss among patients in a coastal city of South India. Indian J Otolaryngol Head Neck Surg.

[12] Dalton DS, Cruickshanks KJ, Klein R, Klein BE, Wiley TL (1998). Association of NIDDM and hearing loss. Diabetes Care.

[13] Al-Rubeaan K, AlMomani M, AlGethami AK (2021). Hearing loss among patients with type 2 diabetes mellitus: a cross-sectional study. Ann Saudi Med.

[14] Lee YY, Kim YJ, Gil ES, Kim H, Jang JH, Choung YH (2020). Type 1 diabetes induces hearing loss: functional and histological findings in an Akita mouse model. Biomedicines.

[15] Kang KW, Pangeni R, Park J, Lee J, Yi E (2020). Selective loss of calretinin-poor cochlear afferent nerve fibers in streptozotocin-induced hyperglycemic mice. J Nanosci Nanotechnol.

[16] Galaviz KI, Narayan KM, Lobelo F, Weber MB (2018). Lifestyle and the prevention of type 2 diabetes: a status report. Am J Lifestyle Med.

